# Inflammation and oxidative stress are associated with major adverse cardiovascular events in adults with preclinical hypertension

**DOI:** 10.1038/s41598-025-25460-z

**Published:** 2025-11-24

**Authors:** Colin J. Gimblet, Dariya Kozlova, Linder H. Wendt, Sadaf Akbari, Mingyao Sun, Patrick Ten Eyck, Steven Crowley, Diana I. Jalal

**Affiliations:** 1 Division of Nephrology, Department of Internal Medicine, Carver College of Medicine, University of Iowa, 200 Hawkins Drive, Iowa City, IA 52242 USA; 2 Institute for Clinical and Translational Science, University of Iowa, Iowa City, IA USA; 3 Division of Nephrology, Department of Medicine, Duke University and Durham VA Medical Centers, Durham, NC USA; 4 Iowa City Veterans Affairs Health Care System, Iowa City, IA USA

**Keywords:** Hypertension, Inflammation, Oxidative stress, Cardiovascular events, Hypertension, Risk factors, Predictive markers

## Abstract

Inflammation and oxidative stress increase with rising blood pressure. Adults with preclinical hypertension (systolic 120–129 mmHg) have a heightened risk of major adverse cardiovascular events (MACE). However, it remains unclear whether inflammation and oxidative stress contribute to MACE risk in this population. We conducted an observational study involving 5405 adults in the Framingham Offspring and Generation III cohorts. Exclusion criteria were anti-hypertensive therapy, history of cardiovascular disease, estimated glomerular filtration rate below 60 ml/min/1.73 m^2^, and type-2 diabetes. Blood pressure categories were defined using the American Heart Association blood pressure criteria as normal, elevated (preclinical hypertension), stage 1, and stage 2. MACE was a composite outcome of incident coronary artery disease, stroke, and all-cause mortality. C-reactive protein, interleukin-6, monocyte chemoattractant protein-1, intracellular adhesion molecule-1, fibrinogen, P-selectin, tumor necrosis factor-alpha receptor 2, lipoprotein-associated phospholipase A2 mass and activity, osteoprotegerin, and urinary isoprostanes increased across blood pressure categories (*P* < 0.001). In stepwise-selected Cox proportional hazards models, a 10-year increase in age (HR [95% CI], 3.46 [2.66,4.51]; *P* < 0.001), current smoking (HR [95% CI], 2.17 [1.08,4.39]; *P* = 0.030), a 10 mg/dL increase in low-density lipoprotein (HR [95% CI], 1.17 [1.09,1.27]; *P* < 0.001), interleukin-6 (HR [95% CI], 1.62 [1.25, 2.09]; *P* < 0.001), and urinary isoprostanes (HR [95% CI], 1.39 [1.03, 1.88]; *P* = 0.033) were associated with higher MACE, while female sex (HR [95% CI], 0.58 [0.37,0.95]; *P* = 0.028) was associated with lower MACE among adults with preclinical hypertension. Interleukin-6 and urinary isoprostanes are associated with MACE in adults with preclinical hypertension.

**Supplementary Information:**

The online version contains supplementary material available at 10.1038/s41598-025-25460-z.

## Introduction

Hypertension affects nearly half of adults worldwide and is a major driver of morbidity and mortality through its strong association with major adverse cardiovascular events (MACE)^1^. The American Heart Association (AHA) and American College of Cardiology (ACC) define hypertension as a systolic blood pressure ≥ 130 mmHg or a diastolic blood pressure ≥ 80 mmHg^2^. However, accumulating evidence indicates that MACE risk begins to rise before the onset of hypertension^2^. Indeed, adults with preclinical hypertension (systolic blood pressure of 120–129 mmHg) exhibit a heightened MACE risk^3–5^, suggesting a continuum of cardiovascular risk even below clinical hypertension thresholds.

Chronic inflammation and oxidative stress have emerged as central underlying mechanisms contributing to MACE through their detrimental effects on cardiovascular structure and function^6–9^. These pathophysiological processes accelerate in tandem with rising blood pressure, as evidenced by a linear relation between higher blood pressure and elevated biomarkers of inflammation and oxidative stress^10–14^. Although inflammation and oxidative stress are linked to MACE in meta-analyses and case–control studies of adults with varying cardiovascular risk, including those with hypertension^15–18^, their association in adults with preclinical hypertension remains poorly defined.

In this study, we sought to characterize inflammation and oxidative stress biomarkers across the AHA/ACC blood pressure categories. Additionally, we examined whether these biomarkers were associated with MACE among adults with preclinical hypertension, defined as the AHA/ACC elevated blood pressure category (systolic 120–129 mmHg and diastolic < 80 mmHg), as well as those with stage 1 and stage 2 hypertension. We hypothesized that biomarkers of inflammation and oxidative stress are increased across blood pressure categories and associate with MACE among adults with preclinical hypertension.

## Results

### Clinical characteristics

**Fig. 1:**
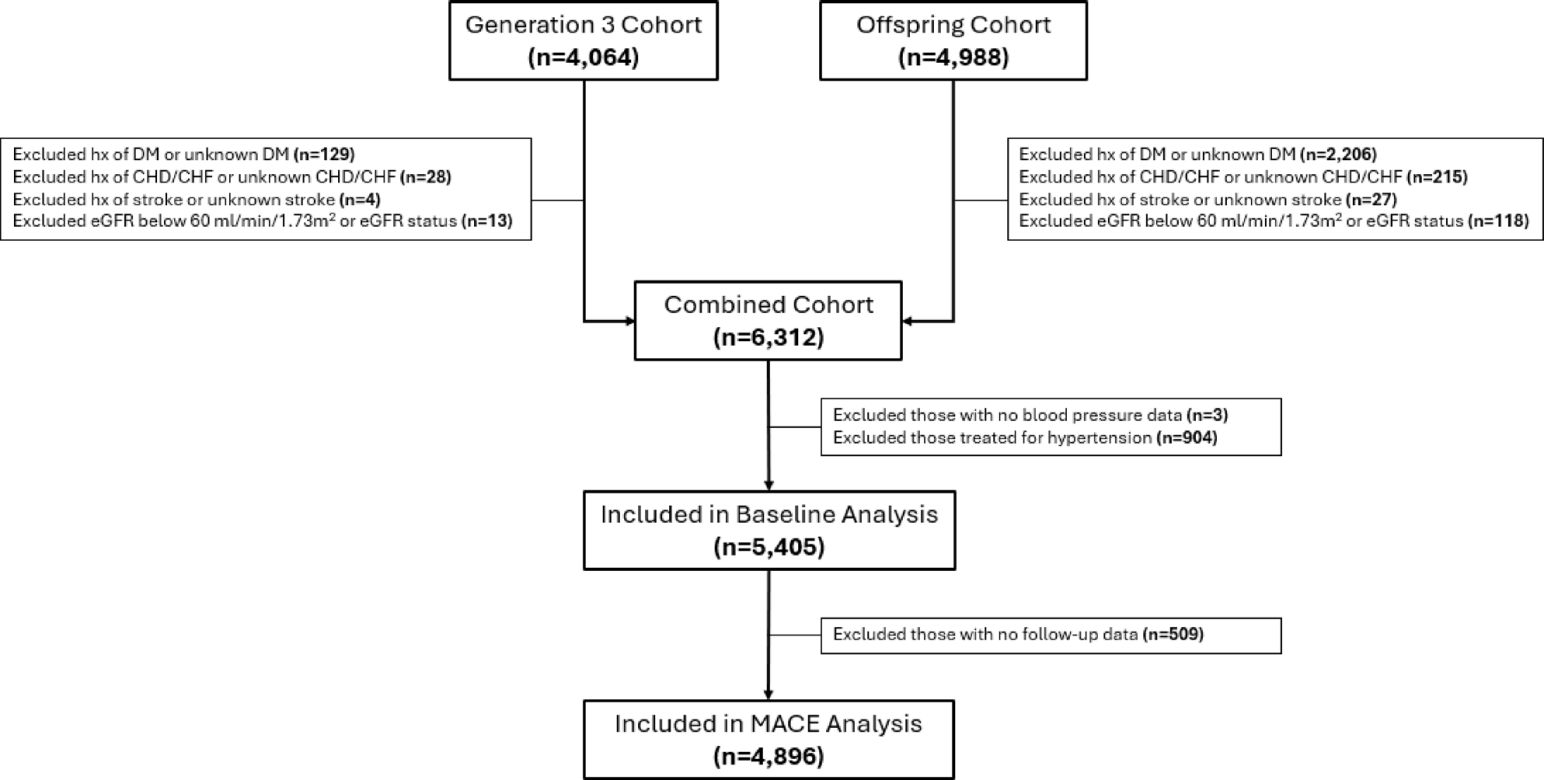
CONSORT diagram for study. DM, diabetes mellitus; CHD, coronary heart disease; CHF, congestive heart failure; eGFR, estimated glomerular filtration rate; MACE, major adverse cardiovascular event; hx, history.

Figure 1 illustrates the study design/consort.

We evaluated the association between biomarkers of inflammation and blood pressure categories in 5405 participants at baseline. The clinical characteristics for those included in the baseline analysis are detailed in Table 1. Participants were predominantly middle-aged and almost entirely white, with a higher proportion of women in the normal blood pressure category and more men in the preclinical, stage 1, and stage 2 hypertension categories. Roughly half of the participants reported no history of smoking, while the remainder were categorized as former or current smokers. Individuals with normal blood pressure had a healthy body mass index, whereas those with preclinical, stage 1, and stage 2 hypertension were primarily classified as overweight. Fasting blood glucose and lipids progressively increased across blood pressure categories, while high-density lipoprotein levels declined. Of note, the use of lipid-lowering medication became more prevalent as blood pressure rose.

**Table 1:** Clinical characteristics.

Variable	Normal (n = 2909)	Elevated (n = 623)	Stage 1 (n = 1274)	Stage 2 (n = 599)	*P* value
Age, years	43 ± 12	49 ± 13	47 ± 12	53 ± 13	< 0.001
*Sex, n (%)*					
Female	1912 (66)	308 (49)	519 (41)	254 (42)	< 0.001
Male	997 (34)	315 (51)	755 (59)	345 (58)	
*Race, n (%)*					
White	2839 (99)	592 (99)	1224 (100)	552(99)	0.40
*Smoking, n (%)*					
Never	1538 (53)	283 (45)	636 (50)	292 (48)	< 0.001
Former	909 (31)	246 (40)	448 (35)	225 (38)	
Current	462 (16)	94 (15)	190 (15)	82 (14)	
BMI, kg/m^2^	25.2 ± 4.3	27.2 ± 4.7	28.3 ± 5.3	29.4 ± 5.7	< 0.001
Systolic BP, mmHg	107 ± 8	124 ± 3	126 ± 8	146 ± 14	< 0.001
Diastolic BP, mmHg	69 ± 7	73 ± 5	82 ± 5	88 ± 10	< 0.001
Fasting glucose, mg/dL	92 ± 8	95 ± 9	96 ± 9	99 ± 9	< 0.001
Total cholesterol, mg/dL	187 ± 35	199 ± 35	199 ± 35	209 ± 42	< 0.001
Triglycerides, mg/dL	97 ± 61	119 ± 87	133 ± 86	153 ± 121	< 0.001
LDL, mg/dL	110 ± 31	121 ± 31	121 ± 33	126 ± 34	< 0.001
HDL, mg/dL	56 (46, 67)	52 (43, 64)	49 (41, 61)	50 (40, 64)	< 0.001
BA-FMD, % dilation	5.9 ± 3.6	4.7 ± 3.7	4.5 ± 3.5	3.2 ± 3.1	< 0.001
cfPWV, m/s	7.18 ± 1.86	8.56 ± 2.63	8.44 ± 2.50	9.87 ± 3.64	< 0.001
Lipid-lowering medication, n (%)	122 (4.2)	45 (7.2)	98 (7.7)	58 (9.7)	< 0.001

Data are presented as mean ± standard deviation for normal variables, median (interquartile range) for all other continuous variables, and count (percentage of participants). One-way ANOVA was used for normally distributed variables, Kruskal–Wallis tests for the non-normal continuous variables, Pearson’s Chi-Square test was used for all categorical variables, except race for which Fisher’s Exact Test was used because of the small numbers of non-white participants. BMI, body mass index; BP, blood pressure; LDL, low-density lipoprotein; HDL, high-density lipoprotein; BA-FMD, brachial artery flow-mediated dilation; cfPWV, carotid-femoral pulse wave velocity.

### Biomarkers of inflammation and oxidative stress

Systemic biomarkers of inflammation, such as C-reactive protein, interleukin-6, MCP-1, TNF receptor-2, fibrinogen, and osteoprotegerin increased as blood pressure categories progressed. Vascular endothelial inflammatory markers, including ICAM-1, P-selectin, and Lp-PLA_2_ mass and activity, were also significantly greater with higher blood pressure levels. Furthermore, urinary isoprostanes, a key indicator of oxidative stress, displayed a similar pattern. In addition, utilizing the Jonckheere-Terpstra test, we found that all the biomarkers had a positive trend with increasing BP categories. Notably, all the biomarkers had a *p* value < 0.0001 except for osteopontegrin (*p* = 0.0019) and TNF receptor-2 (*p* value = 0.0002).

**Fig. 2:**
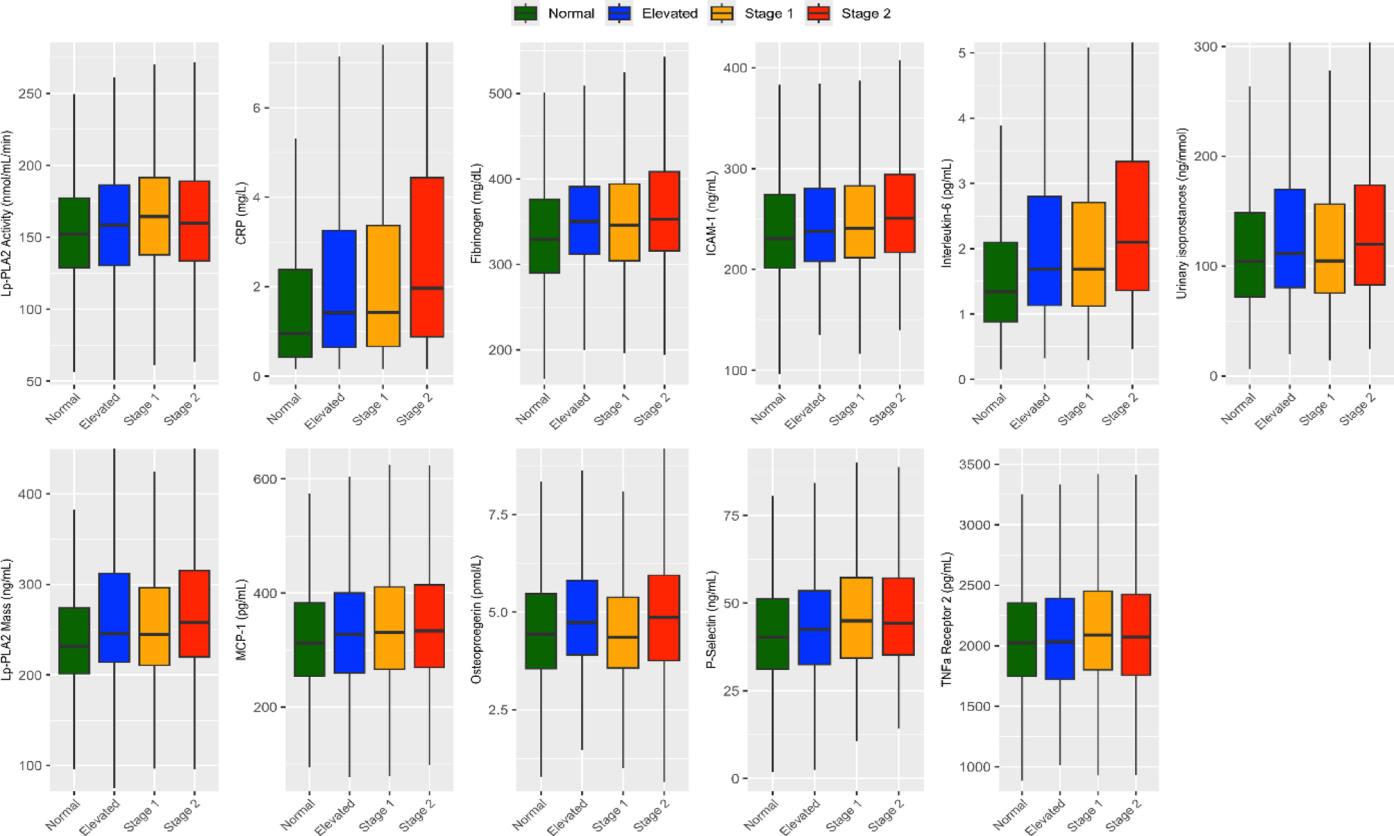
Biomarkers of inflammation and oxidative stress according to the American Heart Association blood pressure categories. Data are presented as median (interquartile range). A one-way ANOVA was used to compare normally distributed variables across blood pressure categories, while Kruskal–Wallis tests were applied to non-normally distributed continuous variables. *P* value < 0.001 for all the shown biomarkers. CRP, C-reactive protein; MCP-1, monocyte chemoattractant protein-1; ICAM-1, intracellular adhesion molecule-1; TNF, tumor necrosis factor, Lp-PLA_2,_ lipoprotein-associated phospholipase A_2_.

Figure 2 and Supplemental Table 1 represent inflammation and oxidative stress biomarkers stratified by the AHA/ACC blood pressure categories.

### Major adverse cardiovascular events in adults across blood pressure categories

**Fig. 3:**
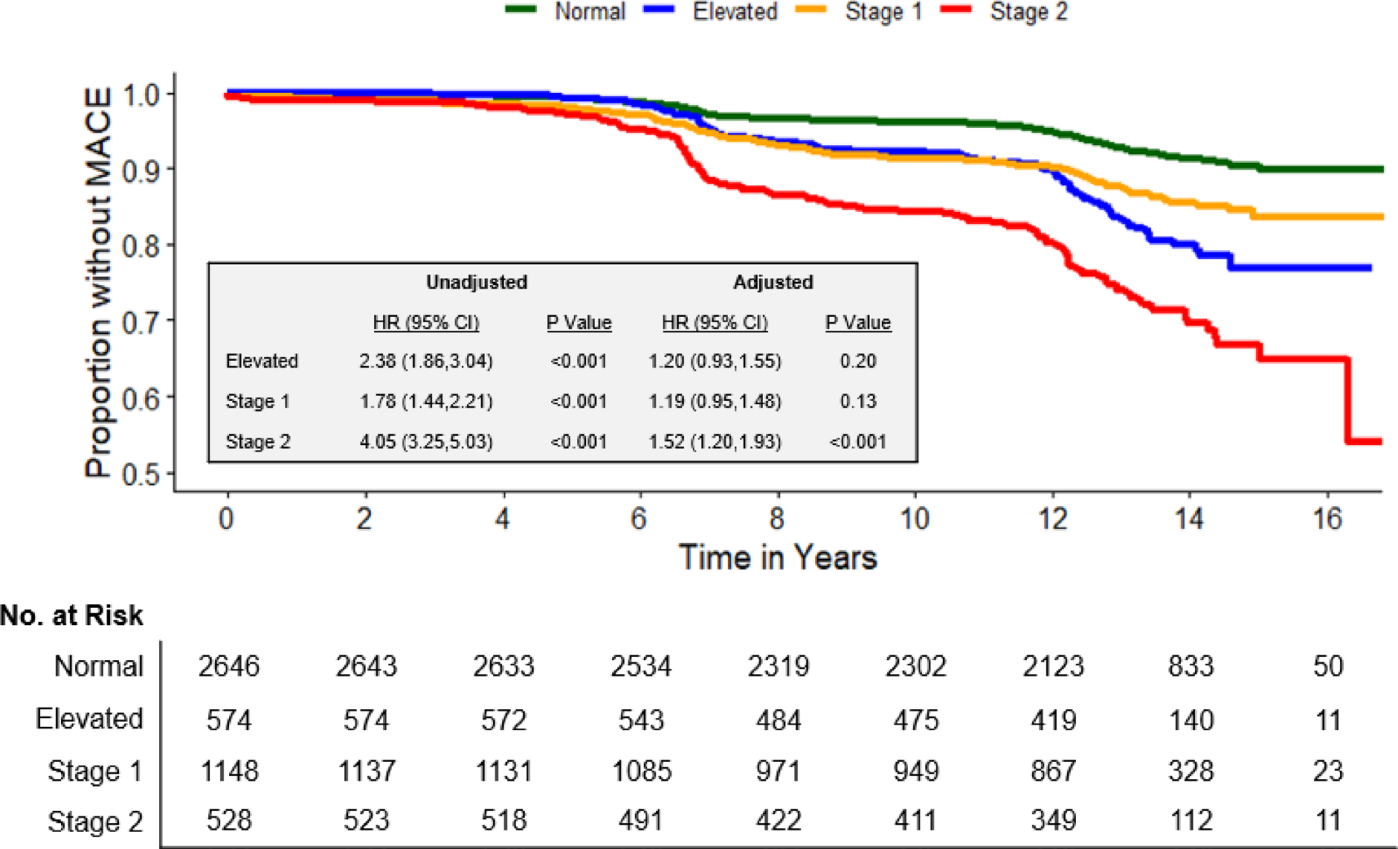
Major adverse cardiovascular events across the AHA/ACC blood pressure categories. Kaplan–Meier survival curves depicting the incidence of major adverse cardiovascular events (MACE) over time across AHA/ACC blood pressure categories. Elevated blood pressure, stage 1 hypertension, and stage 2 hypertension were compared to the normotensive referent group. Hazard ratios (HR) and 95% confidence intervals (CI) were derived from Cox proportional hazard models adjusted for age, sex, body mass index, low-density lipoprotein, and smoking status. MACE, major adverse cardiovascular events; HR, hazard ratio; CI, confidence interval.

Next, we evaluated the association between blood pressure categories and future MACE. This included 4896 participants who had complete follow up data. Baseline characteristics for these participants are shown in Supplemental Table 2. Figure 3 illustrates the proportion of participants free of MACE over time. MACE occurred in 7% (n = 197) of those with normal blood pressure, 16% (n = 93) of those with preclinical hypertension, 13% (n = 145) of those with stage 1 hypertension, and 26% (n = 138) of those with stage 2 hypertension. Median time to event was 10.1 (6.6, 12.4) years in the normal group, 10.9 (6.9, 12.5) years in the preclinical hypertension group, 7.8 (6.2, 12.2) years in the stage 1 group, and 8.2 (6.6, 12.2) years in the stage 2 group.

Before controlling for demographic and clinical characteristics, adults with preclinical hypertension had a 2.38 times greater MACE risk (HR [95% CI] 2.38 [1.86, 3.04]; *P* < 0.001) compared with adults with normal blood pressure. However, after adjusting for traditional cardiovascular risk factors, including age, sex, body mass index, LDL, and smoking status, the significance of this association was abolished (HR [95% CI], 1.20 [0.93,1.55]; *P* = 0.20). Similarly, while stage 1 hypertension showed an increased MACE risk in unadjusted analyses (HR [95% CI] 1.78 [1.44, 2.21]; *P* < 0.001), the significance of this association disappeared after adjustment (HR [95% CI] 1.19 [0.95, 1.48]; *P* = 0.13). In contrast, stage 2 hypertension demonstrated a robust association with MACE both before (HR [95% CI] 4.05 [3.25, 5.03]; *P* < 0.001) and after adjustment (HR [95% CI] 1.52 [1.20, 1.93]; *P* < 0.001). While those in the preclinical hypertension group had a higher rate of events than those in stage 1 hypertension, this was not significant after adjustment for age, sex, and baseline systolic and diastolic blood pressure.

### Predictors of major adverse cardiovascular events within each blood pressure category

Table 2 presents a multivariable model predicting MACE derived through forward stepwise selection across AHA/ACC blood pressure categories. In adults with preclinical hypertension, each decade of advancing age corresponded to a 3.46 times greater risk of MACE. Current smoking conferred a 2.17 times higher MACE risk than never smoking. A 10 mg/dL increase in LDL concentration was associated with a 17% greater MACE risk. Additionally, systemic inflammation, indicated by a doubling of interleukin-6, was associated with a 62% higher MACE risk. Systemic oxidative stress, reflected by a doubling of urinary isoprostane concentration, was linked to a 39% increase in MACE risk. Female sex was associated with a 42% lower MACE risk.

**Table 2:** Multivariate Cox proportional hazards models predicting major adverse cardiovascular events stratified by AHA/ACC blood pressure categories.

Variable	Elevated (n = 484)		Stage 1 (n = 972)		Stage 2 (n = 447)	
	HR (95% CI)	*P* Value	HR (95% CI)	*P* Value	HR (95% CI)	*P* Value
Age, 10-year increase	3.46 (2.66,4.51)	< 0.001	2.40 (1.88,3.08)	< 0.001	2.19 (1.78,2.70)	< 0.001
Female sex	0.58 (0.37,0.95)	0.028			0.64 (0.41,0.99)	0.047
Current smoking	2.17 (1.08,4.39)	0.030	3.77 (2.23,6.35)	< 0.001	2.58 (1.44,4.64)	0.001
Systolic BP, 10 mmHg increase					1.16 (1.01,1.33)	0.039
Diastolic BP, 10 mmHg increase			0.64 (0.47,0.88)	0.005		
Fasting glucose, 10 mg/dL increase			1.21 (1.00,1.48)	0.045		
LDL, 10 mg/dL increase	1.17 (1.09,1.27)	< 0.001				
Log_2_(HDL)					0.53 (0.32,0.88)	0.013
Log_2_(Interleukin-6)	1.62 (1.25,2.09)	< 0.001	1.13 (0.99,1.27)	0.058	1.30 (1.03,1.63)	0.025
Log_2_(MCP-1)			1.21 (1.07,1.37)	0.006		
Log_2_(TNF Receptor-2)			0.00 (0.00,0.07)	0.006		
Log_2_(Lp-PLA_2_ mass)			1.24 (1.04,1.47)	0.016		
Log_2_(Lp-PLA_2_ activity)			1.53 (1.20,1.96)	< 0.001		
Log_2_(Isoprostanes)	1.39 (1.03,1.88)	0.033				

HR, hazard ratio; CI, confidence interval; BP, blood pressure; LDL, low-density lipoprotein, HDL, high-density lipoprotein; MCP-1, monocyte chemoattractant protein-1; TNF; tumor necrosis factor; Lp-PLA_2_, lipoprotein-associated phospholipase A_2_.

In adults with stage 1 hypertension, each decade of advancing age correlated to a 2.40 times greater risk of MACE. Current smoking posed a 3.77 times higher MACE risk than never smoking. A 10 mg/dL increase in fasting glucose was associated with a 21% greater MACE risk. Doubling of MCP-1, TNF receptor-2, and Lp-PLA_2_ mass and activity were linked with a higher MACE risk. A 10 mmHg rise in diastolic blood pressure was associated with a 36% lower MACE risk. Of note, interleukin-6 improved model fit, as reflected by a lower AIC; however, its association with MACE did not reach statistical significance.

In adults with stage 2 hypertension, each decade of advancing age was associated with a 2.19 times greater risk of MACE. Current smokers had a 2.58 times greater risk. A 10 mmHg rise in systolic blood pressure was associated with a 16% increase in MACE risk. Doubling of interleukin-6 was linked to a 30% increase in MACE risk. Doubling of HDL concentration corresponded to a 47% lower MACE risk. Finally, female sex was associated with a 36% lower MACE risk. Importantly, the AIC values for the models with and without the inflammatory biomarkers indicated that the inclusion of the inflammatory biomarkers vastly improved the quality of the model those with elevated BP and stage 1 hypertension, whereas it made little difference for stage 2 hypertension. AIC values were 703, 1322, and 1145 without the inflammatory biomarkers for the categories: elevated BP, stage 1 hypertension, and stage 2 hypertension, respectively. With the inclusion of the inflammatory biomarkers, the A1C values were 688, 1293, and 1142 for elevated BP, stage 1 hypertension, and stage 2 hypertension respectively.

Additional adjustment for BMI, HDL-cholesterol, BA-FMD, cfPWV, and LDL- cholesterol (if not previously selected) was attempted for each of the three multivariate models. In each case, the findings were not substantially altered.

## Discussion

In this study of 5405 adults from the Framingham Offspring and Generation 3 cohorts, we evaluated biomarkers of inflammation and oxidative stress across the AHA/ACC blood pressure categories and their association with MACE. Our findings demonstrate notably higher levels of inflammation and oxidative stress biomarkers with stepwise increases in blood pressure. In addition to age and current smoking, we found an association between inflammation and MACE in those with stage 1 and stage 2 hypertension. Importantly, interleukin-6 and urinary isoprostanes were significantly associated with MACE among adults with preclinical hypertension, alongside traditional risk factors such as age, male sex, current smoking, and LDL. Collectively, these results add to a growing body of evidence demonstrating that higher blood pressure, even in the preclinical stage, is part of the continuum of inflammation, oxidative stress, and cardiovascular disease.

Inflammation and oxidative stress act synergistically within the vasculature and kidneys to drive hypertension and accelerate cardiovascular disease. A primary source of oxidative stress, nicotinamide adenine dinucleotide phosphate (NADPH) oxidase, activates the nuclear factor-κB (NFκB) signaling pathway in the vascular endothelium and kidneys, thereby propagating gene transcription of key inflammatory mediators and exacerbating hypertension^9,19^. Additionally, immune cells and proinflammatory cytokines amplify oxidative stress^20–22^, further elevating blood pressure and reinforcing a vicious cycle of inflammation, oxidative stress, and hypertension. Observational studies consistently show that inflammation and oxidative stress are elevated in adults with a systolic blood pressure ≥ 120 mmHg. For instance, the REGARDS study revealed that interleukin-1β, tumor necrosis factor-α, and interferon-γ are elevated before the onset of hypertension^14^. Additionally, circulating interleukin-6 concentrations are elevated in hypertensive older adults compared with controls^9,12^. Furthermore, greater systolic blood pressure and pulse pressure have been associated with increased urinary isoprostane concentration^8,22^, a widely recognized marker of systemic oxidative stress^23^. Elevated circulating isoprostanes have also been reported in individuals with hypertension^24^. Our study expands upon these findings and identifies several inflammatory and oxidative stress biomarkers that increase as blood pressure rises in adults without prior cardiovascular disease. Together, these findings offer insights into potential mechanisms underlying MACE risk in those with preclinical and clinical hypertension and suggest that cardiovascular risk increases early in the course of disease.

Interleukin-6 is a pro-inflammatory cytokine that plays a pivotal role in mediating the acute-phase inflammatory response and has been directly implicated in the formation and progression of atherosclerotic lesions and coronary heart disease, as demonstrated by a large-scale genetic and biomarker meta-analysis^25,26^. Elevated circulating interleukin-6 levels are reproducibly associated with MACE across diverse populations, including apparently healthy adults, high-risk groups, and those with preexisting cardiovascular disease^16,17,27–29^. Remarkably, this association is homogeneous across all racial and ethnic groups, underscoring its universal role in cardiovascular disease^30^. Our findings expand upon prior research by demonstrating an association between interleukin-6 and MACE in adults with preclinical hypertension and stage 2 hypertension. Moreover, although interleukin-6 was not significantly associated with MACE in stage 1 hypertension, it did enhance model performance, as evidenced by a lower AIC. Stage 2 hypertension encompassed a broader and more severe systolic blood pressure range, which may explain why systolic pressure was associated with MACE in stage 2 but not in preclinical or stage 1 hypertension. In addition, this may have diminished the relative impact of inflammatory biomarkers in stage 2 hypertension. Of note, interleukin-6 inhibition has emerged as a promising therapeutic strategy for reducing MACE in individuals with established cardiovascular disease^25^. The ZEUS trial, which is evaluating interleukin-6 inhibition with ziltivekimab in patients with cardiovascular disease, chronic kidney disease, and systemic inflammation, is highly anticipated for its potential to reshape cardiovascular risk management. While our findings do not support the use of interleukin-6 inhibitors in adults with preclinical hypertension, they highlight that inflammation is evident early in the course of the disease and suggest the potential value of preventive strategies targeting inflammation to reduce cardiovascular risk in this population.

Isoprostanes are formed through the free radical peroxidation of arachidonic acid and serve as a reliable in vivo marker of oxidative stress^23^. Population-based studies have established an independent association between urinary isoprostanes and cardiovascular disease, as well as cardiovascular mortality, in older German adults and postmenopausal women in the Netherlands^15,18^. Expanding on these findings, our study revealed that urinary isoprostanes are associated with MACE in adults with preclinical hypertension, suggesting that oxidative stress may play a more prominent role in early blood pressure elevation. Interestingly, this association was not observed in adults with clinical hypertension, raising the possibility that different pathophysiologic mechanisms predominate with greater blood pressure.

Our study highlights sex differences in MACE risk among adults with preclinical hypertension. Previous studies have shown that men exhibit a higher prevalence of hypertension than women until approximately 65 years of age^31,32^. After this age, women surpass men in hypertension prevalence, which is likely influenced by changes to sex hormones associated with menopause^33^. Interestingly, results from the SPRINT trial demonstrated that women have a lower cardiovascular risk than men^34,35^. This is further supported by evidence revealing that men are, on average, diagnosed with cardiovascular disease at a younger age than women^36^. Consistent with these findings, we observed that women with preclinical hypertension had a lower risk of MACE compared with men. Notably, the majority of adults in our study were middle-aged, which may partly explain why MACE risk was higher in men than women. Further research is warranted to determine whether post-menopausal women face a greater MACE risk than premenopausal women with preclinical hypertension and to understand the role of sex hormones in modulating cardiovascular outcomes in this population.

A strength of our study was the focus on inflammation and oxidative stress in adults with preclinical hypertension, free from preexisting cardiovascular disease, and not taking anti-hypertensive medication. This approach allowed us to elucidate potential mechanisms driving residual cardiovascular risk in a population where anti-hypertensive therapy is not currently indicated^2^. Another strength of our study was the use of stepwise regression with AIC-based model selection, which allowed us to identify an optimal set of predictors for MACE. This unbiased approach balances model accuracy and simplicity while minimizing overfitting.

Several limitations should be noted. First, the observational nature of our study precludes establishing causality between inflammation and oxidative stress biomarkers with MACE in this population. Next, most participants in our cohort were white, thus limiting the generalizability of our findings to other racial and ethnic groups. The AHA/ACC guidelines emphasize the importance of accounting for racial and ethnic diversity in evaluating and treating hypertension^2^. Finally, we were unable to identify sex-specific risk factors associated with MACE because of the limited number of MACE occurrences in our study.

In conclusion, our study reveals that biomarkers of inflammation and oxidative are increased with elevated blood pressure including among individuals with prehypertension. Notably, interleukin-6 and urinary isoprostanes were significantly associated with MACE in adults with preclinical hypertension (systolic 120–129 mmHg and diastolic < 80 mmHg). These findings suggest the need for preventative strategies to modify inflammation and oxidative stress and to reduce cardiovascular risk among those with preclinical hypertension. Additionally, future studies are needed to elucidate sex-specific risk factors driving cardiovascular disease in adults with preclinical hypertension.

## Methods

### Study design

We conducted an observational analysis using data collected between 1998 and 2019 from the Framingham Offspring and Generation 3 cohorts (n = 5405). Of these, 4896 participants had follow-up data available and were included in the analysis of future MACE (Supplemental Table 2). Detailed descriptions of the Framingham Heart Study cohorts are available elsewhere^37,38^. The de-identified data was obtained via a research materials and data use agreement from BioLINCC. The study was conducted in accordance with federal and institutional guidelines. A waiver of informed consent and the analysis plan were approved by the University of Iowa Institutional Review Board (IRB, ID# 201811756). In the Framingham Offspring cohort, exam 7 was considered the baseline visit, with follow-up data collected at exams 8 and 9. For the Framingham Generation 3 cohort, exam 1 was considered the baseline visit, with follow-up data collected at exams 2 and 3. We excluded those receiving anti-hypertensive therapy and those with a history of cardiovascular disease, chronic kidney disease defined as an estimated glomerular filtration rate (eGFR) below 60 ml/min/1.73m^2^, or type-2 diabetes at baseline. Exclusion criteria were chosen to remove the influence of other clinical conditions that could independently affect cardiovascular risk, hence focusing on primordial prevention strategies.

### Clinical characteristics

Blood pressure categories were defined according to the 2017 AHA/ACC guidelines as normal (systolic < 120 mmHg and diastolic < 80 mmHg), elevated (systolic 120–129 mmHg and diastolic < 80 mmHg), stage 1 (systolic 130–139 mmHg or diastolic 80–89 mmHg), and stage 2 (systolic ≥ 140 mmHg or diastolic ≥ 90 mmHg)^2^. Hereafter, we refer to elevated blood pressure as preclinical hypertension, as pharmacologic therapy is not currently indicated in this population. Participant age, sex, race, cardiovascular disease history, and smoking status were obtained through surveys and a physician-administered medical history examination at the baseline visit within the Framingham Offspring and Generation 3 cohorts. In addition, vitals and laboratory measures, including body mass index, systolic blood pressure, diastolic blood pressure, fasting glucose, total cholesterol, triglycerides, low-density lipoprotein (LDL), high-density lipoprotein, and creatinine, were obtained from baseline visits. Vascular function was assessed non-invasively using brachial artery flow-mediated dilation (BA-FMD) as an index of endothelial function and carotid-femoral pulse wave velocity (cfPWV) as an estimate of aortic stiffness. eGFR was calculated using the 2021 CKD-EPI creatinine equation, and a history of chronic kidney disease was defined as eGFR < 60 at the baseline visit.

### Biomarkers of inflammation and oxidative stress

We evaluated a panel of 10 blood biomarkers representing systemic inflammation (high-sensitivity C-reactive protein, interleukin-6, monocyte chemoattractant protein-1 [MCP-1], tumor necrosis factor [TNF] receptor-2, fibrinogen, and osteoprotegerin), vascular endothelial inflammation (P-selectin, intracellular adhesion molecule-1 [ICAM-1], and lipoprotein-associated phospholipase A_2_ [Lp-PLA_2_] mass and activity), and one urine biomarker reflecting oxidative stress (urinary isoprostanes). These specific inflammatory and oxidative stress biomarkers were available in the Framingham Offspring and Generation 3 cohorts and were selected because they allowed for temporal alignment across the Framingham cohorts. A correlation plot evaluating the correlation between these biomarkers (Supplemental Fig. 1S).

Samples were collected in a fasted state, aliquoted, and stored at −70 °C. Comprehensive descriptions of blood and urine methodologies have been previously described^39^. Briefly, C-reactive protein was quantified using a high-sensitivity assay (BN100 nephelometer, Dade Behring). Interleukin-6, MCP-1, TNF receptor-2, P-selectin, and ICAM-1 were measured using commercially available quantitative ELISA kits (R&D Systems). Fibrinogen was determined using the Clauss method (Diagnostica Stago Reagents). Osteoprotegerin was measured using a quantitative ELISA (Biomedica Gesellschaft mbH). Lp-PLA_2_ activity was evaluated by colorimetric activity and Lp-PLA_2_ mass was determined using a commercially available sandwich ELISA (diaDexus). Frozen urine was assayed for 8-epi-PGF2α isoprostanes (isoprostanes) using a competitive enzyme immunoassay kit (Cayman Chemical). Isoprostanes were then normalized to urine creatinine to account for urine tonicity. The mean intra-assay coefficient of variation was ≤ 7.0 for blood tests and ≤ 9.1 for urine tests.

### Primary outcome

MACE, the primary outcome of our analysis, was a composite endpoint of incident coronary artery disease, stroke, and all-cause mortality^40^. Documented MACEs occurred after the baseline visit. Medical records for all hospitalizations and physician visits related to MACE during follow-up were thoroughly reviewed and adjudicated by a panel of three investigators.

### Statistical analysis

Variables that are normal in distribution are presented as mean ± standard deviation, non-normally distributed as median (interquartile range), and categorical as number (percentage of participants). One-way ANOVA, Kruskal–Wallis, and Fisher’s Exact tests were used to assess for differences in clinical characteristics and inflammation and oxidative stress biomarkers across normal, elevated, stage 1, and stage 2 blood pressure categories. Kaplan–Meier curves were generated to illustrate the incidence of MACE over time across blood pressure groups. Univariate and multivariate Cox proportional hazards models were fit to quantify and test these unadjusted and adjusted between-group risks. Blood pressure category was the sole predictor in the univariate model. A subsequent model was fit, adjusting for age, sex, body mass index, low-density lipoprotein, and smoking status. A complete-case analysis using the Cox proportional hazards model with forward stepwise selection based on the Akaike information criterion (AIC) was utilized to identify the optimal predictor set for time to MACE across blood pressure categories. Candidate variables for the selection procedure included traditional risk factors (age, sex, race, smoking status, prediabetes status, body mass index, waist circumference, systolic blood pressure, diastolic blood pressure, fasting blood glucose, total cholesterol, triglycerides, LDL, high-density lipoprotein, non-high-density lipoprotein cholesterol, creatinine, and eGFR) and biomarkers of inflammation and oxidative stress (C-reactive protein, interleukin-6, MCP-1, TNF receptor-2, P-selectin, ICAM-1, osteoprotegerin, fibrinogen, Lp-PLA_2_ mass and activity, and isoprostanes). The univariate associations for these candidate variables were evaluated in each blood pressure category and are shown in Supplemental Tables 3, 4, and 5. Of note, BA-FMD and cfPWV were considered initially and not selected in the model. Considering this and the high rates of missingness in both variables, these were not included in the final stepwise selection model. Right-skewed variables included as candidates in the multivariate model selection were log-transformed to reduce skewness. For all Cox proportional hazards model predictors, the hazard ratio point estimates and 95% confidence intervals were reported along with* p* values. Significance was assessed at alpha = 0.05 for all testing. All statistical analyses were performed in R version 4.3.2 (R Foundation for Statistical Computing).

## Supplementary Information

Below is the link to the electronic supplementary material.

Supplementary Material 1

## Data Availability

The manuscript does not contain the raw data. To obtain access to the data, investigators should submit requests for the data through BioLINCC, including an abstract, protocol/analysis plan, and IRB approval (expedited). Subsequently, a data use agreement will be completed prior to the release of the de-identified data. The data was made available to our group at no cost once these procedures were followed through: https://biolincc.nhlbi.nih.gov/home/
