# Supplementary material for: Inflammation and oxidative stress are associated with major adverse cardiovascular events in adults with preclinical hypertension
Source: Sci Rep. 2025 Nov 24;15:41587. doi: 10.1038/s41598-025-25460-z (PMC12645037; doi:10.1038/s41598-025-25460-z)

| **Supplemental Table 1.** Biomarkers of inflammation and oxidative stress stratified by the AHA/ACC blood pressure categories. | | | | | |
| --- | --- | --- | --- | --- | --- |
| **Variable** | **Normal**  **(n=2,941)** | **Elevated**  **(n=635)** | **Stage 1**  **(n=1,285)** | **Stage 2**  **(n=616)** | **P Value** |
| CRP, mg/L | 0.97 (0.43, 2.47) | 1.43 (0.65, 3.29) | 1.47 (0.67, 3.39) | 1.95 (0.89, 4.38) | <0.001 |
| Interleukin-6, pg/mL | 1.35 (0.89, 2.13) | 1.70 (1.14, 2.86) | 1.70 (1.12, 2.71) | 2.11 (1.37, 3.34) | <0.001 |
| MCP-1, pg/mL | 313 (254, 385) | 327 (260, 398) | 332 (267, 412) | 331 (268, 413) | <0.001 |
| ICAM-1, ng/mL | 231 (202, 276) | 238 (206, 281) | 241 (212, 284) | 251 (218, 296) | <0.001 |
| Fibrinogen, mg/dL | 339±68 | 354±69 | 354±71 | 366±75 | <0.001 |
| P-Selectin, ng/mL | 40 (31, 51) | 42 (32, 53) | 45 (34, 57) | 44 (35, 58) | <0.001 |
| TNF receptor-2, pg/mL | 2,033 (1,755, 2,364) | 2,046 (1,722, 2,393) | 2,094 (1,805, 2,458) | 2,088 (1,768, 2,441) | <0.001 |
| Lp-PLA_2_ mass, ng/mL | 231 (202, 274) | 245 (214, 311) | 245 (211, 296) | 256 (217, 313) | <0.001 |
| Lp-PLA_2_ activity, nmol/mL/min | 152 (129, 178) | 158 (131, 186) | 165 (139, 192) | 160 (134, 188) | <0.001 |
| Osteoprotegerin, pmol/L | 4.43 (3.52, 5.46) | 4.73 (3.89, 5.78) | 4.36 (3.57, 5.39) | 4.86 (3.76, 5.98) | <0.001 |
| Urinary isoprostanes, ng/mmol | 104 (72, 149) | 112 (80, 170) | 105 (75, 156) | 120 (83, 174) | <0.001 |
| Data are presented as mean ± standard deviation for normal variables and median (interquartile range) for all other continuous variables. A One-way ANOVA was used to compare normally distributed variables across blood pressure categories, while Kruskal-Wallis tests were applied to non-normally distributed continuous variables. CRP, C-reactive protein; MCP-1, monocyte chemoattractant protein-1; ICAM-1, intracellular adhesion molecule-1; TNF; tumor necrosis factor; Lp-PLA_2_, lipoprotein-associated phospholipase A_2_. | | | | | |

| **Supplemental Table 2.** Baseline characteristics for participants including in the cox regression analysis evaluating future MACE | | | | | |
| --- | --- | --- | --- | --- | --- |
| **Variable** | **Normal**  **(n = 2,646)** | **Elevated**  **(n= 574)** | **Stage 1**  **(n=1,148)** | **Stage 2**  **(n = 528)** | **P value** |
| Age, years | 43.1 (11.7) | 49.4 (12.9) | 46.9 (11.7) | 52.4 (12.4) | <0.001 |
| Sex, n (%) |  |  |  |  | <0.001 |
| Female | 1,741 (66) | 284 (49) | 473 (41) | 228 (43) |  |
| Male | 905 (34) | 290 (51) | 675 (59) | 300 (57) |  |
| RACE, n(%)  White | 2,629 (100) | 562 (99) | 1,133 (100) | 516 (99) | 0.4 |
| Smoking, n (%) |  |  |  |  | 0.002 |
| Never | 1,414 (53) | 264 (46) | 579 (50) | 261 (49) |  |
| Former | 835 (32) | 227 (40) | 411 (36) | 200 (38) |  |
| Current | 397 (15) | 83 (14) | 158 (14) | 67 (13) |  |
| BMI, kg/m^2^ | 25.1 (4.3) | 27.3 (4.8) | 28.3 (5.3) | 29.6 (5.8) | <0.001 |
| Systolic BP, mmHg | 107.4 (7.5) | 123.6 (2.8) | 125.4 (8.1) | 145.7 (13.4) | <0.001 |
| Diastolic BP, mmHg | 68.8 (6.5) | 73.2 (5.0) | 81.5 (5.0) | 87.8 (9.2) | <0.001 |
| Fasting glucose, mg/dL | 91.4 (8.1) | 95.0 (8.6) | 95.9 (8.6) | 98.6 (8.9) | <0.001 |
| Total cholesterol, mg/dL | 187 (35) | 200 (35) | 199 (35) | 209 (43) | <0.001 |
| Triglycerides, mg/dL | 96 (59) | 120 (89) | 130 (83) | 154 (125) | <0.001 |
| LDL, mg/dL | 110 (31) | 121 (31) | 121 (32) | 126 (34) | <0.001 |
| HDL, mg/dL | 56  (46, 67) | 53  (43, 64) | 49  (41, 62) | 50  (40, 64) |  |

*Data are presented as mean(standard deviation) for normal variables, median (interquartile range) for all other continuous variables, and count (percentage of participants). One-way ANOVA was used for normally distributed variables, Kruskal-Wallis tests for the non-normal continuous variables, Pearson’s Chi-Square test was used for all categorical variables, except race for which Fisher’s Exact Test was used because of the small numbers of non-white participants. BMI, body mass index; BP, blood pressure; LDL, low-density lipoprotein; HDL, high-density lipoprotein*

**Supplemental Table 3.** Predictors associated with MACE in Elevated Blood Pressure category

| **Characteristic** | **HR** | **95% CI** | **p-value** |
| --- | --- | --- | --- |
| Age, 10 Year Increase | 2.89 | 2.40, 3.48 | <0.001 |
| Sex, female | 0.94 | 0.63, 1.42 | 0.8 |
| White Race | 0.84 | 0.12, 6.06 | 0.9 |
| Smoking status |  |  |  |
| Never | — | — |  |
| Former | 2.20 | 1.38, 3.52 | <0.001 |
| Current | 2.39 | 1.32, 4.31 | 0.004 |
| Pre-Diabetes |  |  |  |
| No | — | — |  |
| Yes | 1.89 | 1.23, 2.89 | 0.004 |
| BMI, Unit Increase (kg/cm^2^) | 1.01 | 0.97, 1.05 | 0.6 |
| Waist Circumference, 10 Unit Increase | 1.55 | 1.07, 2.24 | 0.021 |
| Systolic BP, 10 Unit Increase (mm Hg) | 1.81 | 0.89, 3.66 | 0.10 |
| Diastolic BP, 10 Unit Increase (mm Hg) | 0.48 | 0.34, 0.68 | <0.001 |
| Fasting Glucose, 10 mg/dL Increase | 1.31 | 1.05, 1.63 | 0.017 |
| Total Cholesterol, 10 Unit Increase | 1.12 | 1.07, 1.19 | <0.001 |
| LDL, 10 Unit Increase | 1.16 | 1.09, 1.24 | <0.001 |
| log2(HDL) | 0.80 | 0.49, 1.31 | 0.4 |
| Non-HDL, 10 Unit Increase | 1.13 | 1.07, 1.19 | <0.001 |
| Serum creatinine, mg/dL | 1.60 | 0.45, 5.69 | 0.5 |
| GFR, 10 Unit Increase | 0.63 | 0.55, 0.72 | <0.001 |
| log2(CRP) | 1.19 | 1.05, 1.34 | 0.005 |
| log2(IL-6) | 1.72 | 1.49, 1.99 | <0.001 |
| log2(MCP-1) | 1.10 | 0.75, 1.63 | 0.6 |
| log2(TNF receptor-2) | 0.99 | 0.54, 1.81 | >0.9 |
| log2(P-selectin) | 0.62 | 0.46, 0.83 | 0.001 |
| log2(ICAM-1) | 1.64 | 0.92, 2.93 | 0.092 |
| log2(Osteopontegrin) | 2.49 | 1.74, 3.57 | <0.001 |
| Fibrinogen | 1.01 | 1.00, 1.01 | <0.001 |
| log2(Lp-PLA2 mass) | 3.03 | 1.81, 5.06 | <0.001 |
| log2(Lp-PLA_2_ activity) | 0.66 | 0.39, 1.11 | 0.12 |
| log2(Isoprostanes) | 1.52 | 1.20, 1.92 | <0.001 |

*BMI, body mass index; BP, blood pressure; LDL, low-density lipoprotein; HDL, high-density lipoprotein; GFR, glomerular filtration rate;* CRP, C-reactive protein; MCP-1, monocyte chemoattractant protein-1; ICAM-1, intracellular adhesion molecule-1; TNF; tumor necrosis factor; Lp-PLA_2_, lipoprotein-associated phospholipase A_2_.

**Supplemental Table 4.** Predictors associated with MACE in Stage 1 Hypertension category

| **Characteristic** | **HR** | **95% CI** | **p-value** |
| --- | --- | --- | --- |
| Age, 10 Year Increase | 2.70 | 2.34, 3.11 | <0.001 |
| Sex, female | 0.85 | 0.61, 1.20 | 0.4 |
| White Race | 1,211,046 | 0.00, Inf | >0.9 |
| Smoking status |  |  |  |
| Never | — | — |  |
| Former | 2.32 | 1.58, 3.40 | <0.001 |
| Current | 3.41 | 2.19, 5.31 | <0.001 |
| Pre-Diabetes |  |  |  |
| No | — | — |  |
| Yes | 1.80 | 1.28, 2.53 | <0.001 |
| BMI, Unit Increase (kg/m^2^) | 0.98 | 0.95, 1.02 | 0.3 |
| Waist Circumference, 10 Unit Increase | 1.19 | 0.89, 1.59 | 0.2 |
| Systolic BP,10 Unit Increase (mm Hg) | 1.95 | 1.56, 2.44 | <0.001 |
| Diastolic BP, 10 Unit Increase (mm Hg) | 0.37 | 0.30, 0.45 | <0.001 |
| Fasting Glucose, 10 mg/dL Increase | 1.52 | 1.28, 1.81 | <0.001 |
| Total Cholesterol, 10 Unit Increase | 1.06 | 1.02, 1.11 | 0.006 |
| LDL, 10 Unit Increase | 1.08 | 1.03, 1.13 | 0.002 |
| log2(HDL) | 0.87 | 0.59, 1.28 | 0.5 |
| Non-HDL, 10 Unit Increase | 1.06 | 1.02, 1.11 | 0.003 |
| Serum creatinine, mg/dL | 1.94 | 0.68, 5.53 | 0.2 |
| GFR, 10 Unit Increase | 0.66 | 0.59, 0.73 | <0.001 |
| log2(CRP) | 1.15 | 1.05, 1.27 | 0.003 |
| log2(IL-6) | 1.67 | 1.46, 1.91 | <0.001 |
| log2(MCP-1) | 1.26 | 0.88, 1.81 | 0.2 |
| log2(TNF receptor-2) | 0.85 | 0.53, 1.34 | 0.5 |
| log2(P-selectin) | 0.63 | 0.48, 0.83 | <0.001 |
| log2(ICAM-1) | 1.60 | 1.04, 2.46 | 0.033 |
| log2(Osteopontegrin) | 2.75 | 1.95, 3.89 | <0.001 |
| Fibrinogen | 1.00 | 1.00, 1.01 | <0.001 |
| log2(Lp-PLA2 mass) | 5.17 | 3.38, 7.92 | <0.001 |
| log2(Lp-PLA_2_ activity) | 0.96 | 0.61, 1.52 | 0.9 |
| log2(Isoprostanes) | 1.70 | 1.39, 2.08 | <0.001 |

*BMI, body mass index; BP, blood pressure; LDL, low-density lipoprotein; HDL, high-density lipoprotein; GFR, glomerular filtration rate;* CRP, C-reactive protein; MCP-1, monocyte chemoattractant protein-1; ICAM-1, intracellular adhesion molecule-1; TNF; tumor necrosis factor; Lp-PLA_2_, lipoprotein-associated phospholipase A_2_.

**Supplemental Table 5.** Predictors associated with MACE in Stage 2 Hypertension

| **Characteristic** | **HR** | **95% CI** | **p-value** |
| --- | --- | --- | --- |
| Age, 10 Year Increase | 2.89 | 2.40, 3.48 | <0.001 |
| Sex, female | 0.94 | 0.63, 1.42 | 0.8 |
| White Race | 0.84 | 0.12, 6.06 | 0.9 |
| Smoking status |  |  |  |
| Never | — | — |  |
| Former | 2.20 | 1.38, 3.52 | <0.001 |
| Current | 2.39 | 1.32, 4.31 | 0.004 |
| Pre-Diabetes |  |  |  |
| No | — | — |  |
| Yes | 1.89 | 1.23, 2.89 | 0.004 |
| BMI, Unit Increase (kg/m^2^) | 1.01 | 0.97, 1.05 | 0.6 |
| Waist Circumference, 10 Unit Increase | 1.55 | 1.07, 2.24 | 0.021 |
| Systolic BP,10 Unit Increase (mm Hg) | 1.81 | 0.89, 3.66 | 0.10 |
| Diastolic BP, 10 Unit Increase (mm Hg) | 0.48 | 0.34, 0.68 | <0.001 |
| Fasting Glucose, 10 mg/dL Increase | 1.31 | 1.05, 1.63 | 0.017 |
| Total Cholesterol, 10 Unit Increase | 1.12 | 1.07, 1.19 | <0.001 |
| LDL, 10 Unit Increase | 1.16 | 1.09, 1.24 | <0.001 |
| log2(HDL) | 0.80 | 0.49, 1.31 | 0.4 |
| Non-HDL, 10 Unit Increase | 1.02 | 0.00, 1.05 | 0.3 |
| GFR, 10 Unit Increase | 0.63 | 0.55, 0.72 | <0.001 |
| Serum creatinine, mg/dL | 1.60 | 0.45, 5.69 | 0.5 |
| log2(CRP) | 1.19 | 1.05, 1.34 | 0.005 |
| log2(IL-6) | 1.72 | 1.49, 1.99 | <0.001 |
| log2(MCP-1) | 1.10 | 0.75, 1.63 | 0.6 |
| log2(TNF receptor-2) | 0.99 | 0.54, 1.81 | >0.9 |
| log2(P-selectin) | 0.62 | 0.46, 0.83 | 0.001 |
| log2(ICAM-1) | 1.64 | 0.92, 2.93 | 0.092 |
| log2(Osteopontegrin) | 2.49 | 1.74, 3.57 | <0.001 |
| Fibrinogen | 1.01 | 1.00, 1.01 | <0.001 |
| log2(Lp-PLA2 mass) | 3.03 | 1.81, 5.06 | <0.001 |
| log2(Lp-PLA_2_ activity) | 0.66 | 0.39, 1.11 | 0.12 |
| log2(Isoprostanes) | 1.52 | 1.20, 1.92 | <0.001 |

*BMI, body mass index; BP, blood pressure; LDL, low-density lipoprotein; HDL, high-density lipoprotein; GFR, glomerular filtration rate;* CRP, C-reactive protein; MCP-1, monocyte chemoattractant protein-1; ICAM-1, intracellular adhesion molecule-1; TNF; tumor necrosis factor; Lp-PLA_2_, lipoprotein-associated phospholipase A_2_.

**Supplemental Figure 1. Correlation Plot.** This illustrates the correlation between the biomarkers. Of note, the strongest correlations were observed between C-reactive protein (CRP), interleukin-6, and fibrinogen.


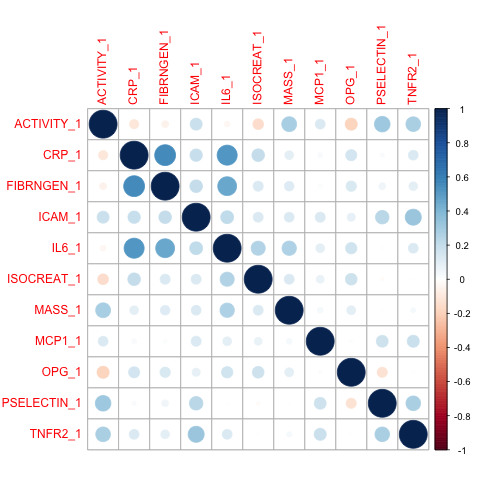

Supplement: Supplementary file 1 — Supplementary Material 1 [file 41598_2025_25460_MOESM1_ESM.docx]
